# COVID-19 Management in the Pediatric Age: Consensus Document of the COVID-19 Working Group in Paediatrics of the Emilia-Romagna Region (RE-CO-Ped), Italy

**DOI:** 10.3390/ijerph18083919

**Published:** 2021-04-08

**Authors:** Susanna Esposito, Federico Marchetti, Marcello Lanari, Fabio Caramelli, Alessandro De Fanti, Gianluca Vergine, Lorenzo Iughetti, Martina Fornaro, Agnese Suppiej, Stefano Zona, Andrea Pession, Giacomo Biasucci, on behalf of the Working Group on COVID-19 in Pediatrics of the Emilia-Romagna Region (RE-CO-Ped)

**Affiliations:** 1 Paediatric Clinic, Department of Medicine and Surgery, University Hospital, 43126 Parma, Italy; 2 Paediatrics and Neonatology Unit, Ravenna Hospital, AUSL Romagna, 48121 Ravenna, Italy; federico.marchetti@auslromagna.it; 3 Paediatric Emergency Unit, Scientific Institute for Research and Healthcare (IRCCS) Azienda Ospedaliero-Universitaria di Bologna, 40138 Bologna, Italy; marcello.lanari@unibo.it; 4 Paediatric Intensive Care Unit, Scientific Institute for Research and Healthcare (IRCCS) Azienda Ospedaliero-Universitaria di Bologna, 40138 Bologna, Italy; fabio.caramelli@aosp.bo.it; 5 Paediatrics Unit, Santa Maria Nuova Hospital, AUSL-IRCCS of Reggio Emilia, 42123 Reggio Emilia, Italy; Alessandro.DeFanti@ausl.re.it; 6 Paediatrics Unit, AUSL Romagna, 47921 Rimini, Italy; gianluca.vergine@auslromagna.it; 7 Paediatric Unit, Department of Medical and Surgical Sciences of Mothers, Children and Adults, University of Modena and Reggio Emilia, 41125 Modena, Italy; lorenzo.iughetti@unimore.it; 8 Paediatrics Unit, G.B. Morgagni—L. Pierantoni, AUSL Romagna, 47121 Forlì, Italy; martina.fornaro@auslromagna.it; 9 Paediatric Clinic, University of Ferrara, 44124 Ferrara, Italy; agnese.suppiej@unife.it; 10 AUSL Modena, 41121 Modena, Italy; s.zona@ausl.mo.it; 11 Paediatric Unit, Scientific Institute for Research and Healthcare (IRCCS) Azienda Ospedaliero-Universitaria di Bologna, 40138 Bologna, Italy; andrea.pession@unibo.it; 12 Paediatrics and Neonatology Unit, Guglielmo da Saliceto Hospital, 29121 Piacenza, Italy; G.Biasucci@ausl.pc.it

**Keywords:** children, COVID-19, MIS-C, pediatrics, SARS-CoV-2

## Abstract

Since December 2019, coronavirus disease (COVID-19) caused by severe acute respiratory syndrome coronavirus 2 (SARS-CoV-2) has rapidly spread, becoming the first pandemic of the 21st century by number of deaths (over 2,000,000 worldwide). Many aspects of SARS-CoV-2 infection in children and adolescents remain unclear, and optimal treatment has not yet been defined. Therefore, our goal was to develop a consensus document, practically synthesizing the accumulated data and clinical experience of our expert group. Literature research was carried out using the keywords “COVID-19” or “SARS-CoV-2” and “children” or “pediatrics” and “prevention” or “diagnosis” or “MIS-C” or “treatment” in electronic databases (MEDLINE, PUBMED), existing guidelines and gray literature. The fact that the majority of the problems posed by SARS-CoV-2 infection in pediatric age do not need hospital care and that, therefore, infected children and adolescents can be managed at home highlights the need for a strengthening of territorial pediatric structures. The sharing of hospitalization and therapeutic management criteria for severe cases between professionals is essential to ensure a fair approach based on the best available knowledge. Moreover, the activity of social and health professionals must also include the description, management and limitation of psychophysical-relational damage resulting from the SARS-CoV-2 pandemic on the health of children and adolescents, whether or not affected by COVID-19. Due to the characteristics of COVID-19 pathology in pediatric age, the importance of strengthening the network between hospital and territorial pediatrics, school, educational, social and family personnel both for strictly clinical management and for the reduction in discomfort, with priority in children of more frail families, represents a priority.

## 1. Introduction

Since December 2019, coronavirus disease (COVID-19) caused by coronavirus 2 severe acute respiratory syndrome (SARS-CoV-2) has rapidly spread, becoming the first pandemic of the 21st century by number of deaths (over 2,000,000 worldwide) [1,2]. Children appear to be less affected than adults, with a milder clinical presentation and a significantly lower mortality rate [3,4,5]. However, serious complications may occur in pediatric age, such as COVID-19 temporary multisystemic inflammatory syndrome (MIS-C) [6]. Many aspects of SARS-CoV-2 infection in children and adolescents remain unclear, and optimal treatment has not yet been defined. Therefore, our goal was to develop a consensus document, synthesizing the accumulated data and clinical experience of the Emilia-Romagna region, Italy.

## 2. Material and Methods

Literature research was carried out using the keywords “COVID-19” or “SARS-CoV-2” and “children” or “pediatrics” and “prevention” or “diagnosis” or “MIS-C” or “treatment” in electronic databases (MEDLINE, PUBMED), existing guidelines and gray literature. A Writing Committee was created, composed of the 12 directors of the pediatric hospital units of the Emilia-Romagna region, Italy, supplemented by infectious diseases experts from the same units, community and primary care pediatricians; consensus on the content was reached after multiple cycles of review among the 12 members of the Writing Committee. The consensus document was subsequently reviewed and modified with experts from the Emilia-Romagna region and further shared with the extended group (RE-CO-Ped Working Group). As this revision is not intended to duplicate existing guidelines, the GRADE methodology was not used, and no formal recommendations are made. A total of 182 publications were selected and on the basis of the literature as well as clinical experience, a practical proposal for the management of COVID-19 in children and adolescents was developed.

## 3. Results

### 3.1. What Are the Preventive Measures to Use?

Current evidence suggests that SARS-CoV-2 spreads from person to person directly and/or indirectly (through contaminated objects or surfaces) through close contact with predominantly symptomatic (but also asymptomatic) infected people through secretions of the mouth and nose (saliva, respiratory secretions or droplets) and perhaps fecal matter [7].

The main differences that characterize pediatric age, compared to adult age, and that must be considered in relation to preventive measures against SARS-CoV-2 are that in children and adolescents, the disease manifests itself in a less severe manner than in adults, with 96% of cases being asymptomatic or with mild-moderate symptoms and a lethality in the age group 0–15 years of 0.08% [8]. Moreover, children are mainly infected within households, while transmission within educational services is rare. Most school-age cases (40%) occurred in adolescents (14 and 18 years old), followed by primary school children aged 6–10 (27%), middle school children aged 11–13 (23%) and 3–5-year-old preschool children (10%) [9,10]. However, the role of children and adolescents in the spread of the infection remains to be clarified with precision [11]. Younger children (preschool and primary) appear to transmit SARS-CoV-2 less often than adolescents. Transmission by adolescents can occur with the same frequency as adults in family and community settings [11]. Little is known about the spread of the infection of the new variants of SARS-CoV-2 [11].

During the pandemic, stressful factors such as lack of social contact and loss of school routines have been associated with long-term neuropsychiatric sequelae. In particular, several studies have found high levels of anxiety, depression and post-traumatic stress disorder in quarantined children [12,13]. For these reasons, among the various interventions of social isolation implemented as preventive measures to contain the epidemic [14], the closure of educational services and schools is the most discussed and considered less relevant one [15], in the face of an important social, behavioral and psychological fallout [16].

The preventive measures currently universally recommended against SARS-CoV-2 transmission and to be adopted even in pediatric age are summarized in Table 1 [17,18,19,20,21].

***Statement:*** Children become infected and transmit SARS-CoV-2 probably less frequently than adolescents and adults. In childhood, COVID-19 is, however, less serious. The same preventive measures apply to children as indicated for the general population. The prolonged closure of schools does not have, on the basis of available data, a relevant effect on the spread of the pandemic, while it is associated with greater stress and discomfort for children and adolescents.

### 3.2. When Should the SARS-CoV-2 Swab Be Performed?

The indication to perform a diagnostic test for SARS-CoV-2 depends on various factors that influence the strategy to be used and that can change over time (e.g., spread of the virus, type of restrictive measures, availability of diagnostic tests). The clinical presentation of COVID-19 in pediatric age is heterogeneous and substantially superimposed on most common viral infections. The main symptoms are fever (59.1%), cough (55.9%), diarrhea (21.4%), nasal congestion and rhinorrhea (20%), pharyngitis (18.2%), asthenia and myalgia (18.7%), dyspnea (11.7%), nausea and vomiting (5.4%) and headache (4.3%) [22,23,24]. Anosmia, hyposmia, ageusia and dysgeusia have been reported less frequently in the pediatric population than in adults. More frequently, however, highly polymorphic skin lesions such as livedo reticularis, purpuric lesions, measles lesions, multiform-like erythema lesions, extremity cyanosis and vesicular lesions have been detected [25]. Rare, but still possible, are heart complications with chest pain and hypoperfusion related to heart failure, arrhythmia, myocarditis and shock, kidney complications up to kidney failure with the need for hemodialysis and systemic complications such as sepsis and MIS-C [26]. Often, gastrointestinal symptoms are not associated with respiratory symptoms.

It is not indicated to perform the nasopharyngeal swab in case of a child with symptoms other than the ones described above or in the presence of another known infectious focus which does not require hospitalization. In practice, in a context of home care or an emergency room, the suspicion of COVID-19 is placed by the pediatrician on the basis of a set of assessments they must take into account: (a) the type of clinical symptoms; (b) whether or not the symptoms can be attributed to other potential causes (e.g., fever without other localization, concomitant otitis, pharyngotonsillitis), it is useful to initiate specific therapy (when indicated) by recommending isolation and observing clinical evolution and recovery; (c) the family and social epidemiological context (positive medical history for symptomatic family member; risk profile of cohabiting family members by age, pathologies and profession; community frequency). The literature agrees on carrying out molecular investigation in the case of a symptomatic child or a close contact child [23], defined as contact with a proven positive subject, in the absence of protective devices, for a time of more than 15 min, at a distance of less than 1 m. It is also recommended to perform the examination in all patients who need to be admitted to hospital (extended screening), also for reasons not related to SARS-CoV-2 (e.g., diagnostic work-up, medical or surgical procedures and/or interventions) given the high prevalence and possible contagiousness of asymptomatic positive children [27]. This precaution is recommended in order to avoid intra-hospital outbreaks, although literature data show very low positivity to screening tests (<1%) and it is reasonable to think that this strategy should change in favor of selective screening, should the epidemiological context change [28]. It is recommended to perform tests for SARS-CoV-2 also in patients with respiratory infection due to respiratory syncytial virus or influenza in consideration of the possibility of co-infection [29]. It is not indicated to perform the nasopharyngeal swab in case of a child with symptoms other than the one described or in the presence of another known infectious focus which does not require hospitalization.

***Statement:*** The swab for the search for SARS-CoV-2 in the pediatric population is recommended in subjects with suspected clinical manifestations of SARS-CoV-2 infection, also taking into account the family and social epidemiological context. It is indicated in asymptomatic children who are close contacts of probable or confirmed positive patients, in patients who require hospitalization and in case of return to Italy from one of the countries defined as at risk. The swab is not recommended in the case of a child with symptoms that have a known infectious focus and who do not need hospitalization.

### 3.3. What Are the Samples with Optimal Sensitivity and Specificity for Diagnosis?

Since the report of the first pediatric case of COVID-19, the molecular biology by means of a real time-polymerase chain reaction (RT-PCR) test on nasopharyngeal (NP) and oropharyngeal (OP) samples has been considered the “gold standard” for the diagnosis of COVID-19 in pediatric age [2,3,4]. The RT-PCR test allows identifying and amplifying viral RNA sequences: SARS-CoV-2 can be found in the biological sample from the week before the onset of symptoms until the second week after the onset of symptomatology, with maximum positivity found in the first week. Detection of the virus depends on a number of factors: the presence of sufficient viral quantities in the sample, collection of the sample carried out in the first week of onset of symptoms, appropriate procedures for collecting, storing and processing the sample [30].

Recent studies on the adult population have shown that SARS-CoV-2 is detected more frequently through NP than OP swabs, suggesting a significantly higher SARS-CoV-2 charge in the NP samples than in the OP samples [30,31]. As stated by experts from the United States Centers for Disease Prevention and Control (CDC), testing strategies and the recommended sample type are the same for children and adults [32]. According to experts from the CDC, NP, OP, middle nasal swirl, nasal aspirate, nasopharyngeal or nasal and salivary swabs are all acceptable samples [32]. Similarly, the American Academy of Pediatrics does not recommend one sample over another [33]. The guidelines of the World Health Organization (WHO) (updated in September 2020) recommend a sample of the upper respiratory tract (NP and/or OP) to correctly diagnose SARS-CoV-2 infection [34].

Recently, alternative samples of the upper respiratory tract have been suggested for the pediatric population. A prospective study comparing nasal and OP swabs in children with COVID-19 showed a significantly higher positivity rate and significantly higher average viral load on nasal samples, supporting nasal sample collection instead of OP [35]. In addition, nasopharyngeal aspirate has been proposed as an alternative to the NP swab [36].

To date, most studies have focused on specific patient groups, and the size of their sample is too small to recommend alternative samples, such as saliva, to detect SARS-CoV-2 infection. While awaiting further and wider studies to determine whether a collection method is more reliable than the others to diagnose COVID-19 in children, guidelines and experts agree that NP and OP swabs are optimal samples in pediatric age.

***Statement:*** NP and OP swabs are usually used to diagnose SARS-CoV-2 infection in children: the NP, among those of the upper respiratory tract, appears to be the most sensitive. Molecular testing (RT-PCR) is also considered the gold standard for children and adolescents.

### 3.4. After How Many Days Should the Swab Be Repeated in Case of Positive SARS-CoV-2 Result?

Collecting a sample of the upper respiratory tract in a child to detect SARS-CoV-2 is often challenging and stressful due to the well-known discomfort with the procedure and poor collaboration of children. Currently, according to international guidelines, a second test is not required to confirm the presence of SARS-CoV-2, both in adults and children, after the completion of the quarantine period and the resolution of symptoms [37,38].

The duration of the isolation period is uncertain and depends on the persistence of viral elimination. Recommendations on isolation times in pediatric age are similar to those in adults. The exact duration of infectivity of COVID-19 patients is not yet known for certain. Several studies have shown that it is greater at the onset of symptoms and that SARS-CoV-2 can be detected in samples taken from the upper respiratory tract about two days before the onset of symptoms [39]. In non-severe case studies, the virus was detected up to 10 days after the onset of symptoms [39]. Among hospitalized patients with severe clinical manifestations, SARS-CoV-2 was detected until the twentieth day after the onset of symptoms, with an eight-day median value [39].

The WHO initially recommended confirming the elimination of the virus and ending isolation after performing two negative RT-PCR swabs, taken at least 24 h apart [40]. Currently, WHO criteria allow isolation to be stopped without requiring a new specific test for SARS-CoV-2 after 10 days after the onset of symptoms for symptomatic patients, as well as at least three more days without symptoms [41]. In the case of asymptomatic patients, the WHO requires isolation for at least 10 days after the last SARS-CoV-2-positive test [41]. The European Centre for Prevention and Disease Control (ECDC) recommends stopping isolation on the basis of criteria that take into account the clinical resolution of symptoms, the time elapsed since their onset, the severity of the disease, the immune status and the evidence of viral RNA clearance from the upper respiratory tract. In the event that isolation times are respected, the control swab may not be executed; otherwise, there is a need for two consecutive swabs 24 h apart [42]. In Italy, the Ministry of Health expressed the need for a negative control swab in asymptomatic people who have previously tested positive for SARS-CoV-2, after a period of isolation of at least 10 days [43]. The control swab is also necessary in symptomatic patients and in close contacts who cannot respect an adequate period of isolation from the appearance of symptoms [43]. In other cases, it is possible to end the isolation without carrying out a swab [43]. However, the recommendations for these change rapidly and are not necessarily standardized across countries.

The updated criteria to free COVID-19 patients from isolation are mainly due to awareness of limited laboratory supplies, equipment and personnel in areas with intense viral transmission. In addition, there is growing evidence that the prolonged presence of viral RNA does not necessarily imply infectivity. A recent study conducted in children with COVID-19 showed an average viral spread duration of about 11 and 23 days from the onset of symptoms through the respiratory tract and gastrointestinal tract, respectively [44]. A study on virus culture and contact traceability suggested that patients with mild to moderate COVID-19 are highly unlikely to be infectious beyond 10 days after the onset of symptoms [45].

***Statement:*** The recommendations on isolation times in children are similar to those of adults. In particular, (1) the asymptomatic positive child/adolescent can end isolation after at least 10 days from the onset of positivity, at the end of which a molecular test with a negative result is performed; (2) the symptomatic positive child/adolescent can end isolation after at least 10 days from the onset of symptoms and if they perform a molecular test with negative results after at least 3 days without symptoms; (3) in the event of persistent positivity and in the absence of symptoms for at least a week, the patient can finish isolation after 21 days from the onset of symptoms; (4) close contacts of SARS-CoV-2-infected cases must observe either a quarantine period of 14 days from the last exposure to the case or a quarantine period of 10 days from the last exposure with a negative antigen or molecular test performed on the tenth day; (5) the child/teenager returning from a country at risk, in addition to a negative test carried out in the 48 h prior to entry into Italy, must observe a 14-day quarantine period.

### 3.5. What Is the Usefulness of Molecular, Antigenic and Serological Tests?

Considering the evidence currently available, an appropriate choice of diagnostic tests to be used for SARS-CoV-2 is essential. Among the criteria to be considered in the choice of tests, it is necessary to evaluate not only the sensitivity and specificity of the individual methods but also the execution times, the need for an available laboratory and the costs. Currently, molecular tests are available on NP or OP swabs, and antigenic tests on nasal swabs, NP, OP or salivary samples, besides serological tests. There are no different indications for the pediatric and adult populations.

Molecular tests consist of an RT-PCR that detects parts of the SARS-CoV-2 virus (RNA fragments) [46,47]. This method is the gold standard for diagnosis: the result can be obtained in 3–5 h, and sensitivity is almost 100%, but trained personnel, specific reagents and expensive equipment are required.

Rapid antigen tests, on the other hand, are direct, qualitative assays, which evaluate the presence of the virus in the biological sample, containing as substrate a specific antibody capable of binding with SARS-CoV-2 viral antigens, and the result can be visible to the naked eye without the need for a laboratory [48,49,50]. They are faster and cheaper than gold standard tests, but less sensitive. In particular, the sensitivity is 70–86% and the specificity is 95–97%. Rapid antigen tests require a sample containing thousands of viral particles per microliter to give a positive result. The test may be negative if the concentration of antigens is below the detection limit (e.g., test performed too early with respect to exposure or too late with respect to the onset of symptoms). Therefore, these tests are especially useful for screening investigations, such as those within schools [48,49], where a low pre-test probability (low prevalence) results in a high negative predictive value. Conversely, the positive predictive value under pre-test low-probability conditions implies a high false positive rate. It follows that a positive antigenic test carried out in the screening environment must necessarily be confirmed with a second NF/OF swab analyzed via molecular biology, with the risk of increasing the discomfort especially for children <6 years [50].

Serological tests, on the other hand, provide indirect information, i.e., they detect the presence of specific antibodies against the more immunogenic proteins of SARS-CoV-2, namely, the spike protein (S) and/or nucleoprotein (N), and indicate a previous or ongoing infection [51]. The specificity of these tests varies between 96.5% and 100% for automated testing and between 94.7% and 96.5% for rapid immune-enzymatic tests, while the overall sensitivity varies between 63.2% and 73.1%, with optimal performance four weeks after symptoms [52]. They are mainly useful for detecting exposure to the virus and therefore also used in epidemiological seroprevalence studies. In case of positivity, it is necessary to re-evaluate the patient by means of a molecular test to exclude the potential residual contagiousness.

On the basis of actual Italian recommendations and clinical experience of the panel of experts, the usefulness and limitations of antigen and serological tests in pediatric age are summarized in Table 2.

***Statement:*** Swab RT-PCR is the gold standard for the diagnosis of SARS-CoV-2. Rapid anti-gene tests are faster and cheaper than molecular but less sensitive and are especially useful for screening investigations in the school setting. Serological tests, on the other hand, provide indirect information and are useful in the epidemiological field in seroprevalence studies.

### 3.6. When Should the SARS-CoV-2-Positive Pediatric Patient Be Hospitalized?

The WHO classification, taken up by the Canadian Pediatric Society [53], identifies disease severity degrees, subdividing the clinical pictures as follows: (1) asymptomatic-mild, in case of the presence of fever and/or asthenia and/or symptoms compatible with upper respiratory infection without respiratory distress and/or instrumental evidence of pneumonia; (2) moderate, in the presence of respiratory distress and/or reduced nutrition and hydration and/or instrumental evidence of pneumonia; (3) severe, in the presence of severe respiratory distress and/or desaturation (SpO_2_ < 92% in ambient air) and/or intermittent cyanosis or apnea and/or systemic symptoms such as lethargy, dehydration, convulsions or suspected sepsis; (4) critical, if acute respiratory distress syndrome (ARDS), multiorgan failure (MOF), septic shock or coma occurs.

Most of the positive SARS-CoV-2 pediatric patients present with an asymptomatic-mild clinical picture. The decision on whether to hospitalize a positive child is essentially based on three factors: severity of the disease, comorbidity and family compliance. The evidence for this in the literature is still limited, and, in fact, the percentages of admission of positive subjects in pediatric age vary in different cases from 2% to 60% [5,54,55,56]. A recent study carried out in Emilia-Romagna region, Italy, focused on the importance that adequate territorial management has on reducing the rate of hospitalization of these children [57]. Territorial management was performed in the so-called Community Health Houses that are health structures able to perform nasopharyngeal swabs for SARS-CoV-2 identification, send samples to the laboratory, collect results and organize adequate home monitoring of children infected with SARS-CoV-2 [57].

There is unanimous agreement on the indication for hospitalization in moderate to critical forms and in feverish infants under 3 months of age. In neonatal severe forms or prolonged forms in infancy, primary immunodeficiency must be suspected and excluded, based on recent data demonstrating mutations of Toll-like receptor 3 and type 1 interferon in patients with severe SARS-CoV-2 pneumonia [58].

On the basis of panel experience, children with mild symptoms can be managed at home with telephone surveillance and, when indicated, possible home visit by the family pediatrician or by the Pediatric Special Units for Continuity of Care (USCAP), after caregivers’ education on clinical signs of deterioration (such as respiratory distress, chest pain, cyanosis, alteration of consciousness, oliguria). If the family compliance is inadequate, hospitalization is indicated. Children with chronic diseases, especially chronic lung, heart, neuromuscular and oncological diseases and immunodeficiency disorders, are a heterogeneous group with a potential higher risk of developing a severe disease, although reported cases are few and the overall risk remains low [5,56,57]. In these cases, hospitalization is recommended, although, especially in asymptomatic-mild forms, home management with caregiver supervision is reasonable.

***Statement:*** Hospitalization is recommended in case of moderate to severe illness, in the febrile infant less than 3 months of age and in case of poor family compliance. In case of severe underlying chronic disease (pulmonary, heart, neuromuscular and oncological diseases, immune deficiencies), hospitalization is recommended. In the asymptomatic-mild forms, home management with supervision by the caregiver is reasonable.

### 3.7. What Are the Main Recommendations Other than Routine Ones to Follow in SARS-CoV-2-Positive Pediatric Patients with Chronic Disease?

Overall, children with chronic disease are a high-risk population from an infectious point of view and, therefore, may be prone to develop more severe COVID-19 [59]. In this context, data are still scarce, but the available experience allows some guidance to be drawn on the management of these patients. Of utmost relevance is the recommendation to educate children and their families on preventive behaviors: social distancing, use of suitable masks, also considering the size, and hygiene of the hands, as well as adequate nutrition, proper exercise and regular sleep. In the most acute epidemic phases, it is better to reduce clinical controls to those strictly necessary using telemedicine resources, but ensuring direct and timely contact if symptoms of suspicion, especially respiratory or gastrointestinal, appear [60]. The vaccination schedule, including seasonal influenza vaccination, must continue unchanged [61]. There is no indication to modify, reduce or suspend usual therapies without a specific clinical indication. The following are specific indications for certain categories of patients on the basis of panel experience and literature review. Other recommendations have been provided for further chronic diseases, but they were not considered in this consensus document.

Immunocompromised patients (inflammatory bowel disease, chronic liver disease, chronic kidney disease, organ transplantation, rheumatological disease). The risk of developing a severe form of COVID-19 is low, probably due to the protective effect of immunosuppressive drugs that reduce the inflammatory response [62,63,64,65]. According to specific guidelines, usual therapies, including biological therapies, should be continued. In the presence of COVID-19-related moderate-severe respiratory symptoms, hospitalization and early identification of any signs of pneumonia are indicated. COVID-19-specific therapies should take into account underlying diseases (e.g., glomerular filtration in case of chronic renal failure), and the remodeling of immunosuppressive therapies should be considered on the basis of the severity of the infection and the type of drugs used [66]. If discontinued, immunosuppressive therapy should be resumed after two weeks in asymptomatic patients or after complete clinical resolution in symptomatic patients.

Patients with chronic respiratory diseases. Moderate-severe bronchial asthma is a risk factor for morbidity and mortality from COVID-19 and, therefore, optimal control of symptoms is necessary through appropriate therapies according to guidelines [67]. Metered-dose inhalers or dry powder should be preferred to sprayed drugs that increase the risk of spreading the virus. Acute exacerbations of asthma should be treated promptly and, if necessary, also with the use of corticosteroids. In severe asthma, biological drugs should not be suspended, except during the possible acute phase of COVID-19. Patients with cystic fibrosis, although at risk of respiratory infections, do not show a higher prevalence of COVID-19 than the general population [68]. Some factors could act as protective: the habit of hygienic and preventive measures, the use of drugs and the altered inflammatory pulmonary response.

Patients with hemato-oncological diseases. There is no higher incidence of infections in these patients than in the general population [69]. Therefore, by respecting the rules of hygiene and distancing and by arranging dedicated areas and personnel for positive or suspicious subjects, it is possible to guarantee the access of outpatient patients during therapy or for urgent visits. It is still controversial whether chemotherapy should be delayed in asymptomatic SARS-CoV-2-positive patients.

Patients with neuromuscular diseases. As in the case of other respiratory infections, particularly flu, the risk of respiratory failure during COVID-19 [70] should be mentioned in these patients. Prevention rules, the continuation of pharmacological therapies and respiratory physiotherapy and attention to the appearance of respiratory symptoms are recommended.

Patients with endocrinological diseases. The most challenging endocrinological conditions in COVID-19 are type 1 diabetes (T1D) and adrenal insufficiency (AI). Patients with T1D are poorly represented among those hospitalized for COVID-19 [71,72] and T1D does not appear to predispose to SARS-CoV-2 infection in pediatric and adolescent ages [73]. It is recommended, however, to maintain a good glycemic control to avoid complications in case of infection. As it has been clearly shown [74], telemedicine plays an important role, allowing regular contact with patients to be maintained and to improve metabolic control even during quarantine. The fear of COVID-19 could interfere with a timely diagnosis of T1D and cause an increase in severe diabetic ketoacidosis episodes [73]. Therefore, it is extremely important that family pediatricians do not hesitate to refer a child with suspected onset of diabetes to a specialized center so as to promptly carry out the appropriate diagnostic tests [75].

Although AI patients have a high risk of infection and the chronic glucocorticoid requirements may expose them to a higher risk of viral disease [76], no increase in the COVID-19 rate in AI patients has been reported to date. However, considering that infection is an acute stressful condition requiring a higher dose of glucocorticoids, and adrenal crises during infections are the leading cause of death in patients with AI [77], in the event of COVID-19 symptoms, a rapid increase in the glucocorticoid regimen should be promptly initiated. Thus, in both Addison’s disease and congenital adrenal hyperplasia, whenever presenting with fever and respiratory symptoms, the glucocorticoid dose should be immediately doubled [78]. Furthermore, it is important to advise patients to take abundant amounts of electrolyte-containing fluids. Finally, pediatricians should keep in mind that if these patients present with vomiting or diarrhea, they should be admitted to hospital immediately.

***Statement:*** In children with chronicity, there is no higher incidence or severity of COVID-19. However, it is appropriate to reinforce the preventive messages addressed to these children and their families. In the acute epidemic phases, it is recommended to reduce clinical controls to those strictly necessary using telemedicine resources, but guaranteeing direct and timely contact if symptoms of suspected COVID-19 appear, especially of the respiratory or gastrointestinal type. Vaccination against seasonal influenza is recommended in these patients. There are no indications to modify, reduce or discontinue the usual therapies without a specific clinical indication.

### 3.8. What Are the Symptoms and Signs to Make MIS-C Diagnosis Possible?

MIS-C is a hyperinflammatory syndrome that follows exposure to SARS-CoV-2 of 2–6 weeks and affects children and adolescents [79,80,81]. This syndrome may have clinical-laboratory characteristics similar to other entities such as Kawasaki’s disease, toxic shock syndrome and macrophage activation syndrome [79,81,82,83]. There are various definitions of MIS-C proposed by the WHO, the Royal College of Pediatrics and Child Health (RCPCH) and the CDC (Table 3) [79,80,84], which share the presence of persistent fever (>38 °C), a systemic inflammatory state with an elevation of inflammatory indices, neutrophil leukocytosis, lymphopenia and the presence of organ dysfunctions (Table 4), together with laboratory or epidemiological evidence of COVID-19 and the exclusion of other microbiological causes [79,80,84]. Unlike the adult, in the pediatric patient, the respiratory system is rarely affected (4.5%) with non-specific symptoms such as cough or pharyngitis; much more frequent (>70%) and severe are gastrointestinal diseases, presenting with enteritis up to acute abdomen symptoms, and cardiovascular complications with myopericarditis, hypotension and shock, with the need for cardiovascular support in intensive care unit in 77% of cases [85]. Cardiac involvement manifestations may be an increase in enzymes (troponin and pro-BNP in 80.9% and 84.9% of cases, respectively) and/or left ventricular dysfunction (45%), valve failure (mitral and aortic), pericarditis and arrhythmias [82,83,86,87,88,89,90,91,92]. Coronary dilations or aneurysms in the acute phase of the disease are documented in 6–24% of cases [82,83,87,88,89,90,93,94,95,96,97,98,99,100,101]: they are generally of a non-severe degree and decline in most cases [102]. Given the systemic nature of the syndrome, mucocutaneous interest with rash, conjunctivitis and peripheral edema is also frequent [82,103,104,105]. The RE-C Working Group follows the MIS-C classification suggested by the WHO.

***Statement:*** MIS-C is a hyperinflammatory syndrome that follows exposure to SARS-CoV-2 for 2–6 weeks. Symptoms and signs for making a diagnosis are represented by persistent fever (>38 °C), systemic inflammatory state with elevation of the indexes of inflammation, neutrophilic leukocytosis, lymphopenia and organ dysfunction, together with laboratory or epidemiological evidence of infection by SARS-CoV-2 and exclusion of other microbiological causes.

### 3.9. When Is Only Symptomatic Treatment Indicated in COVID-19?

COVID-19 in children occurs, in most cases, in a paucisymptomatic way or with mild symptoms that do not require hospitalization. In patients below the age of 18 years with COVID-19, cough, pharyngodynia, fever, rhinorrhea, vomiting, diarrhea and headache are the most common symptoms that may deserve a treatment. As the patients’ age increases, fever becomes less common. Basically, these are non-specific symptoms and common to any viral infection. More rarely, there may be breathing difficulty which must be assessed as to whether or not hospitalization is needed. Symptomatic treatment is the same as that used for common respiratory infections and gastroenteritis. In particular, the use of paracetamol is indicated for fever and pain [106,107,108,109]. With regard to the use of nonsteroidal anti-inflammatory drugs (NSAIDs; in particular, ibuprofen), which was initially the subject of controversy [109], on the basis of the results of observational studies, both the European Medicine Agency (EMA) and the WHO do not recommend their possible use [106,107,108,109]. Anyway, patients who already regularly take NSAIDs should not interrupt them. As for any drug, benefits and risks of known adverse events should be evaluated on an individual basis and prescribed accordingly, taking into account indications for its use. In particular, the use of NSAIDs is contraindicated in the presence of a state of dehydration for the increased risk of kidney failure [109].

For respiratory symptoms that may benefit from inhalation therapy with steroids and bronchodilators, spacer devices are preferred over nebulizer ones to reduce the spread of viral particles in the air [54,110]. Finally, in case of diarrhea or vomiting, proper hydration with oral rehydrating solutions should be ensured.

Antibiotics are not indicated except in cases of bacterial superinfection. Recent studies in adult patients have documented the wide and improper use of antibiotic therapy which could be a significant cause of increased bacterial resistance [111]. The number of cases with a co-infection or bacterial complication is not known in pediatric age. In the adult patient, it turns out to be extremely low. A meta-analysis that estimated the risk of a concomitant bacterial infection in adult patients with COVID-19 documented that bacterial co-infection (at the onset of symptomatology) was present in 3.5% of cases and secondary bacterial infection in 14.3% [112]. The overall percentage of COVID-19 patients with bacterial infection is 6.9%. Bacterial infection is most common in critical patients (8.1%). Most patients with COVID-19 received antibiotics (72%) [54].

On the basis of the current knowledge in adult patients, azithromycin is not indicated for use for therapeutic, immunomodulatory or antiviral purposes (as initially hypothesized) in COVID-19, both in the early stages of infection and in the most severe stages [113,114].

***Statement:*** In the majority of symptomatic cases of SARS-CoV-2 infection in children and adolescents, only symptomatic therapy with paracetamol or possibly with ibuprofen is recommended after excluding a state of dehydration. In the case of respiratory symptoms that may benefit from inhalation therapy with bronchodilators and/or cortisones, a spacer is preferred over a nebulizer to reduce the spread of viral particles in the air. In case of diarrhea or vomiting, proper hydration should be ensured with oral rehydrating solutions. Antibiotic therapy is not indicated except in the presence of a likely bacterial complication. The use of azithromycin is not indicated.

### 3.10. Are Immunomodulatory Drugs in COVID-19 in Pediatric Age Indicated?

In children with moderate-severe clinical conditions, with pneumonia and progression in deterioration of respiratory function, acute respiratory distress syndrome (ARDS) or clinical conditions that are part of the diagnosis of MIS-C, the addition of immunomodulatory therapy should be considered to support therapy [115,116,117,118].

In particular, the use of corticosteroids is recommended by WHO guidelines for adults with COVID-19 and with moderate-severe clinical conditions. Steroid therapy in these patients reduces mortality and improves the clinical course [115,116,117,118]. In children with lung infection requiring oxygen supplementation, studies are still ongoing and the benefits/risks are still uncertain.

In case of serious clinical conditions or in case of rapid clinical deterioration with regard to respiratory symptoms (SpO2 < 92%), referring to the adult model, low-dose glucocorticoids may be administered for a duration of 10 days or until discharge, depending on which is the shortest duration [118]. However, there is currently no unanimous agreement as to what the optimal duration of therapy should be, which should therefore be assessed on a case-by-case basis [119]. The steroids that can be used are dexamethasone (0.15–0.4 mg/kg/day per os/iv, maximum dose 6 mg), prednisone (1–2 mg/kg/day per os, maximum dose 60 mg) or methylprednisolone (1–2 mg/kg/day iv, maximum dose 60 mg) [54,115,118]. In cases of severe MIS-C (see specific paragraph), the use of high-dose methylprednisolone (10–30 mg/kg/day, maximum dose 1 gr, for 3–5 days) may be envisaged [119].

Based on the results of controlled clinical trials in the adult population, tocilizumab, a monoclonal antibody directed against interleukin (IL)-6 receptors, is not indicated in moderate-severe forms of respiratory failure [120,121]. The use of anakinra, an IL-1 inhibitor, should be considered in second-line treatment in cases of MIS-C (see specific paragraph) and in cases of ARDS [122].

The monoclonal antibody bamlanivimab, directed against the SARS-CoV-2 S protein, would act in the early stages of the disease by blocking the attack of the virus and therefore should be indicated in mild-moderate forms of infection. A randomized Phase II placebo-controlled study demonstrated the effectiveness of the drug in reducing viral load and in improving clinical symptomatology by reducing the use of hospitalization [123]. The Food and Drug Administration (FDA) also authorized its use in pre-adolescents and non-hospitalized adolescents aged ≥12 years and weighing ≥40 kg, with mild-moderate disease and with risk factors for the development of a serious disease (e.g., heart disease, sickle cell anemia, chronic respiratory disease). It should be administered within 10 days of the onset of symptoms and at the single iv dose of 700 mg over 60 min [124].

Regarding other immunomodulating therapies that have been proposed, the available evidence in adult patients does not support hydroxychloroquine therapy, either for preventive [125,126] or therapeutical use, alone or in combination with azithromycin [126,127].

The efficacy of interferon [128] and donor plasma [129] is not yet proven and their use in pediatric age is not recommended unless in clinical trials that are ongoing.

***Statement:*** In children with severe clinical conditions, with pneumonia and progression to deterioration of respiratory function, acute respiratory distress syndrome (ARDS) or clinical conditions that are part of the diagnosis of MIS-C, the addition of immunomodulatory therapy should be considered as supportive care. In case of severe clinical conditions or in case of rapid clinical worsening of respiratory symptoms (SpO_2_ < 92%), low-dose glucocorticoids can be administered for a duration of 10 days or until discharge, whichever is the shortest duration. The use of anakinra can be taken into consideration in the second-line treatment in cases of MIS-C and in cases with a picture of ARDS, even in the absence of evidence of efficacy and preferably in the context of clinical studies. The use of monoclonal antibodies in the initial stages of the disease can be considered in the context of clinical studies and specific clinical indications. The use of tocilizumab is not recommended and there is no evidence to support hydroxychloroquine therapy for either preventive or therapeutic use alone or in combination with azithromycin, and the efficacy of interferon or plasma has not been demonstrated in donors.

### 3.11. When Is Anticoagulant Therapy Indicated and with What Drugs in COVID-19 in Pediatric Age?

COVID-19 is associated with a hypercoagulation state and disseminated intravascular coagulation due to the cytokine storm unleashed during severe disease [130,131,132]. There is increasing evidence to suggest that the set of immune dysregulation, endothelitis and hypercoagulation state results in microvascular thrombosis in pulmonary parenchyma, with consequent severe respiratory failure. Some guidelines, therefore, recommend the prophylactic use of low-molecular weight heparin in all hospitalized adult patients, with an intermediate dose in adult patients admitted to intensive care and the use of anticoagulant therapy in patients with suspected thrombosis [133].

The incidence of thromboembolic events during COVID-19 in children is not known, due to the low incidence of severe disease in pediatric age [133]. An American registry shows an incidence of thromboembolic events of 7% (3/45) among patients aged 13–21 years and 1.3% (1/75) between 5 and 13 years of age [134]. All children who presented with COVID-19-related ongoing thromboembolic events had more than one risk factor for thrombosis [133].

A recent American consensus suggests starting antithrombotic prophylaxis in children hospitalized with severe disease (see paragraph on MIS-C), who have at least one risk factor for thrombosis or a high blood concentration of D-dimer (≥5 times normal value), in the absence of contraindications. Prophylaxis is recommended with low-molecular weight heparin at low dose two times a day (target of the level of anti-Xa activity 4 h after administration from 0.2 to 0.5 U/mL) or non-fractional heparin in continuous infusion (target of anti-Xa activity level from 0.1 to 0.35 U/mL). Antithrombotic prophylaxis is not recommended in the presence of thrombocytopenia with platelet count <20,000–50,000/μL, hypofibrinogenemia <100 mg/dL, recent major bleeding and acetylsalicylate therapy at a dose of >5 mg/kg/day. Anti-thrombotic prophylaxis is indicated after discharge for a further period to be assessed (e.g., another 30 days) in case of D-dimer elevation at discharge or persistence of risk factors for thrombosis. Antithrombotic prophylaxis is not routinely indicated in all children hospitalized for COVID-19.

***Statement:*** Antithrombotic prophylaxis with heparin is recommended in hospitalized children with severe disease, who have at least one risk factor for thrombosis or a high blood dose of D-dimer (≥5 times the normal value), in the absence of contraindications.

### 3.12. When Is Antiviral Therapy Indicated and with What Drugs?

Antiviral therapy for COVID-19 is not necessary for the vast majority of pediatric patients and is recommended for severe and critical cases, as defined above [54,135,136,137].

Among the antivirals, remdesivir is suggested, preferably as part of a clinical trial. Remdesivir binds to viral RNA-dependent RNA polymerase, inhibiting viral replication through premature termination of RNA transcription and demonstrating in vitro activity against SARS-CoV-2 [138,139]. The recommended dosage is: in children weighing ≥40 kg, 200 mg iv over 30 min on Day 1, followed by 100 mg iv/day for another 9 days; in children weighing <40 kg, 5 mg/kg iv over 30 min on Day 1, followed by 2.5 mg/kg iv (over 30 min)/day for a further 9 days. At present, the dosage has not been established for the first 2 weeks of life [139]. Therapy can be continued for 10 days, although a duration of 5 days is appropriate for most patients [139].

The safety and efficacy of remdesivir in association with corticosteroids have not been properly studied to date; however, there are theoretical reasons why a combined therapy may be useful in some patients with severe COVID-19 [140]. Remdesivir, although well tolerated, can cause gastrointestinal symptoms (e.g., nausea), hypersensitivity reactions, high levels of liver transaminases and an increased prothrombin time.

The association of lopinavir/ritonavir, an enhanced protease inhibitor used in HIV infection therapy, from the age of 14 days of life [140], should be used only in the context of clinical studies, given the lack of efficacy reported in the literature.

The antiviral role of hydroxychloroquine, which seems to act by increasing the endosomal pH required for virus/host cell fusion and interfering with SARS-CoV-2 cell receptor glycosylation [141,142], is not documented and conclusive data from the ongoing trial are needed to understand the possible role of this drug in children with COVID-19 [143].

***Statement:*** Antiviral therapy for COVID-19 with remdesivir is unnecessary for the vast majority of pediatric patients; for serious and critical cases, it can be considered even in the absence of clear efficacy data. The drug is available through an AIFA web-based registry from 12 years of age and 40 kg of weight, and below that age and weight as part of a program of compassionate use or within approved clinical trials. Lopinavir/ritonavir or hydroxychloroquine therapy is not recommended.

### 3.13. What Therapy Is Recommended in MIS-C?

Lacking shared guidelines on the topic, recommendations on therapeutical management are based on experience in managing patients with MIS-C and information available for other pediatric conditions with similar characteristics (Kawasaki disease model, KD). Two consensus documents on MIS-C management are currently available: one from a group of 98 British experts [144] and the other from the American College of Rheumatology (ACR) [145]. On thromboembolic prophylaxis, the Consensus of the Pediatric/Neonatal Hemostasis and Thrombosis Subcommittee of the ISTH SSC has been published [134].

The goal of treating MIS-C is to reduce systemic inflammation and restore organ functions. Two phenotypic models are distinguished: a KD-like model, clinically similar to KD, and a picture without a specific phenotype but which falls within the MIS-C classification criteria. The therapeutical strategy is based on three aspects: 1) immunomodulatory treatment of inflammation, 2) support measures (shock treatment, antibiotic therapy, gastroprotection); 3) management of the pro-coagulation state.

#### 3.13.1. Immunomodulatory Treatment

Immunomodulatory treatment consists in the use of immunoglobulin, corticosteroids or biological drugs (Table 5).

Considering phenotypic overlap with KD, first-line anti-inflammatory therapy involves the administration of i.v. immunoglobulin (IVIG) at a dose of 2 g/Kg. A second dose of IVIG is provided in cases not responding or partly responding to the first infusion [146,147,148,149,150,151,152].

The British consensus recommends the use of high-dose corticosteroids in addition to IVIG only in patients with high-risk KD-like conditions according to the KD protocol [144,149,150], while the consensus of the ACR recommends the combination of IVIG and steroids at high doses for critical patients and at low doses in other cases [144]. Due to the emergence of resistance to treatment with IVIG only in patients with MIS-C [82], the combination of low-dose steroids and IVIG could be considered as the first-line treatment in all cases of non-critical MIS-C [150].

Biological drugs (anakinra, tocilizumab, infliximab) are used as a third line of therapy in cases not responding to the second dose of IVIG and steroid boluses [144,145]. However, in patients with severe MIS-C, they can be administered, according to clinical judgment, as first-line treatment [149,150,151,152,153,154,155,156,157,158]. The British consensus [144] does not indicate a preferred choice among the three drugs; due to safety reasons, the ACR [146] refers to anakinra as the drug of choice in pediatric patients with hyperinflammatory syndromes and active infection, according to the experience on a limited number of MIS-C patients reported in the literature [90,146,153,155].

#### 3.13.2. Support Measures

Support measures are shown in Table 6. Patients in shock require cardiovascular support with fluids and inotropic drugs; in case of ventricular dysfunction, extracorporeal circulation is used [146]. It is recommended to use proton pump inhibitors in case of concomitant steroid therapy and the initiation of i.v. antibiotic therapy in cases presenting with a septic-like picture, until the results of microbiological tests [144].

#### 3.13.3. Pro-Coagulation Status Management

MIS-C results in a pro-coagulation state, with a significant increase in D-dimer and fibrinogen [130,131,134]. Acetylsalicylic acid is recommended at antiaggregating dosage (3–5 mg/kg/day) in KD-like and/or heart involvement and/or thrombocytosis [145,159].

The American consensus [134] suggests starting antithrombotic prophylaxis in children hospitalized with severe disease who have at least one risk factor for thrombosis or an elevated blood D-dimer concentration (≥5 times normal value), in the absence of specific contraindications (Table 7). The stratification of MIS-C patients for thromboembolic risk in relation to D-dimer is shown in Table 8. In patients with unstable or kidney damage, prophylactic heparin continuous infusion is recommended [134]. Antithrombotic prophylaxis is not recommended in the presence of thrombocytopenia (platelet count < 20,000–50,000/μL), hypofibrinogenemia (<100 mg/dL) or recent major bleeding [134]; it is indicated after discharge for a further period to be assessed (e.g., further 30 days) in case of persistence of high D-dimer or persistence of risk factors for thrombosis [134].

***Statement:*** The goal of MIS-C treatment is to reduce systemic inflammation and restore organ function and is based on immunomodulatory treatment of inflammation (immunoglobulins, corticosteroids, biologics), supportive measures (shock treatment, antibiotic therapy, gastroprotection) and management of the pro-coagulation state.

### 3.14. When Should SARS-CoV-2-Positive Pediatric Patients be Admitted into Intensive Care Unit?

The criteria for admission to a pediatric intensive care unit (PICU) for the SARS-CoV-2-positive patient are not different from the general admission criteria for non-positive pediatric patients [160,161,162,163,164,165,166]. In addition, it will also be necessary to ensure a specific intensive track that ensures proper isolation, as well as the adoption of the appropriate safety devices by patients and their caregivers, in order to avoid dangerous cross-infections between patients and patients, and between patients and health care workers, according to international standards redefined on the basis of local policies [167].

A SARS-CoV-2-positive child may require hospitalization into a PICU for: (1) the direct consequences of viral infection, generally severe respiratory failure; (2) complications of infection (e.g., MIS-C); (3) the onset, in conjunction with COVID-19, of other conditions that give indication to the hospitalization. The admittance to a PICU is indicated in cases of severe respiratory failure as in the case of severe respiratory distress according to the WHO definition (tachypnea associated with at least one of the following signs or symptoms: cyanosis, hypoxia, inability to feed and drink, lethargy, unconsciousness or convulsions) and/or ARDS, and, finally, in case of the need for continuous monitoring of the effectiveness of non-invasive respiratory care [168,169]. If intubation is needed, transfer to a PICU is unavoidable. Other indications for hospitalization into a PICU are cardiovascular instability not responding to the volemic filling and/or requiring amine administration. It should be remembered that particularly in the pediatric patient, hypotension is a late sign of insufficiency and that pediatric septic shock after the initial *“resuscitation bundle”* (*golden hour)* requires advanced multimodal monitoring [170,171].

The hemodynamic aspect is of utmost importance in the evaluation of the patient with ascertained or presumed MIS-C. In moderate or severe forms, the marked predisposition to rapid deterioration of cardiovascular function and the complex clinical picture, variously characterized by hemodynamic instability, respiratory impairment, alteration of neurological state, signs of dehydration, signs of acute kidney failure and severe abdominal pain, may require hospitalization in an intensive environment. In this regard, the presence of left ventricular dysfunction, coronary abnormality or pericardial effusion, in addition to alterations of laboratory tests (C-reactive protein, procalcitonin, IL-6, ferritin, D-dimer, troponin, liver transaminases, lactate dehydrogenase) and the rapidity of their modifications over time, can facilitate the choice of the most appropriate care setting [84,166,172].

***Statement:*** The PICU admission criteria do not differ between positive or negative pediatric patients for SARS-CoV-2 infection and are represented by the direct consequences of the viral infection (generally severe respiratory failure), by the complications of the infection (e.g., MIS-C) and by the onset, concomitantly with SARS-CoV-2 infection, of other conditions for which hospitalization is indicated.

### 3.15. How Should the Critical SARS-CoV-2-Positive Pediatric Patient Be Managed?

Pediatric patients admitted to a PICU are assisted by specialized personnel with a nurse/patient numerical ratio able to provide optimal treatment and monitoring. The therapy to overcome organ failure and concomitant pathologies is combined with proper nutritional support, pain management and prevention of infections. In this regard, patients should be managed preferentially in negative pressure rooms, with at least 12 air changes per hour. In patients treated for concomitant diseases, treatment should not be suspended but possibly modified according to the evolution of the overall clinical picture and to the risk/benefit ratio related to the new infectious condition.

Given the different epidemiology compared to the adult patient, and the paucity of data available, it is not possible to give specific therapeutic indications for the pediatric SARS-CoV-2-positive patient. The intensive treatment of respiratory failure, however, follows the progressive graduality criteria indicated by internationally validated guidelines [173]. In the event of failure of high-flow oxygenation (HFNC) techniques, continuous positive airway pressure (CPAP), non-invasive ventilation (NIV) and invasive ventilation by endotracheal intubation (IT) should be carried out. NIV techniques are preferred over HFNC by some experts [174], while others question this approach aimed at increasing alveolar recruitment [175].

Close attention must be paid to the protection of personnel, especially in maneuvering or using techniques at risk of aerosols generation that require second-level protection devices (PPE). In this regard, data from adult patient studies seem to significantly minimize the HFNC-related risk, especially when combined with the use of a facial mask, compared to a high-flow ventimask [176].

Intubation is a high-risk aerosol-generating procedure and must be carried out using suitable PPE and by experienced personnel. It is strongly recommended, associated, where possible, with a transparent cover (to avoid the dispersion of the aerosol), to make use of the video laryngoscope [175], thus increasing the distance between the patient and the operator and reducing the number of unsuccessful attempts; the use of cuffed endotracheal tubes and of a closed-circuit suction system to minimize aerosol generation during bronchoaspiration is advisable [176].

The patient should be pre-oxygenated with O_2_ 100% and it is strongly recommended to make use of the rapid intubation sequence (RSI). General guidelines for the ventilation of pediatric patients with ARDS can be applied to SARS-CoV-2 patients [177]. It is advisable to maintain a tidal volume of 4–6 mL/Kg of ideal weight and the choice of PEEP should be titrated according to the clinical picture and the patient’s ventilator parameters. These patients benefit from pronation [178]. In nonresponsive cases, it is possible to consider the use of nitric oxide (iNO) and high-frequency oscillatory ventilation (HFOV). In case of shock or ARDS refractory to therapy, following strict selection criteria, extracorporeal membrane oxygenation (ECMO) should be considered.

In case of hemodynamic instability, with low blood pressure and signs of poor perfusion and dehydration, it is important to provide volemic filling with balanced crystalloid solutions (10–20 mL/Kg in repeated bolus up to a maximum of 40–60 mL/Kg during the first hour). Especially in case of respiratory failure, it is advisable to use fluid responsiveness indices and advanced hemodynamic monitoring to better guide the hemodynamic support without exceeding the water overload. In case of the need for amine support, epinephrine and norepinephrine are the first-line treatment [179], depending on whether or not cardiac systolic dysfunction is present. As second-line drugs, milrinone, levosimendan and dobutamine should be considered. In cases of nonresponsive shock, the use of vasopressin and glucocorticoids should be evaluated. In the critical patient or in suspected MIS-C, cardiological assessment and, if necessary, advanced hemodynamic monitoring should be carried out. In case of signs of myocarditis, the use of IVIG should be considered. It is recommended to consider the use of subcutaneous enoxaparin prophylaxis, especially in patients at high risk of thrombosis. In thrombocytopenia-associated multiple organ failure (TAMOF) where platelet count reduction reflects a microvascular thrombotic process that can lead to ischemia and organ dysfunction including acute renal failure, plasma exchange treatment may be useful.

***Statement:*** Supportive therapy for organ failure and treatment of concomitant diseases must be integrated with treatment aimed at satisfying water–nutritional needs, minimizing suffering, and preventing infections and other complications. In the patient undergoing therapy for concomitant pathologies, the therapy should not be suspended but possibly modified based on the evolution of the overall clinical picture and the risk/benefit ratio linked to the new infectious condition.

### 3.16. With Respect to Mental Health, Is There a Need for Support Interventions for COVID-19-Positive Children and Adolescents?

Most children and adolescents who test positive for SARS-CoV-2 are, as mentioned, asymptomatic or paucisymptomatic. However, little is known about the possible consequences of the infection, often affecting the parents, on their overall state of mental health, also considering the required home isolation or the rare need for hospitalization. In the literature, emotional and overall discomfort problems resulting from the lockdown period, the school closure and poor socialization are becoming increasingly evident also in the pediatric population. The results of the studies show that the nature and extent of this impact depend on various vulnerability factors such as developmental age, pre-existing physical and mental health conditions, being economically disadvantaged, but also being quarantined due to infection or risk of infection [16,180,181,182].

***Statement:*** It is the duty of health professionals in charge of assisting children and families positive to COVID-19 to ensure adequate follow-up and overall support, with reassurance and rapid reintegration within the school community (also in the case of teaching at a distance) and social community. It is necessary to detect early signs of a possible emotional distress, even family, ensuring, if necessary, support that involves different professional figures (pediatrics, neuropsychiatry, social and educational-scholastic world).

## 4. Conclusions

This consensus document summarizes current knowledge—mostly based on expert consensus and retrospective primary observational studies—and experiences on the management of SARS-CoV-2 infection in children and adolescents with an aim to share principles of good practice. We think that our statements can be useful across Italy as well as European countries with a similar epidemiology. Although local experience elsewhere may vary, some of our positions have been included among the documents of good clinical practice of the Italian Minister of Health [183]. Given the rapid evolution of the pandemic, it will be useful to update its contents based on the production of new evidence. The salient aspects of the document are highlighted below:

- The sharing of hospitalization and therapeutic management criteria for severe cases between professionals is essential to ensure a fair approach based on the best available knowledge;
- The prophylactic use of antibiotics in the management of pediatric patients with COVID-19 is not associated with advantages in terms of morbidity and mortality and contributes to the development of antimicrobial resistance;
- The activity of social and health professionals must also include the description, management and limitation of psychophysical-relational damage resulting from the SARS-CoV-2 pandemic on the health of children and adolescents, whether or not affected by COVID -19, and must be able to inform and alert at the political level;
- Due to the characteristics of COVID-19 pathology in pediatric age, the importance of strengthening the network between hospital and territorial pediatrics (both at the level of free choice pediatrics and at the level of community pediatrics and child neuropsychiatry and adolescents), school, educational, social and family personnel both for strictly clinical management and for the reduction in discomfort, with priority in children of more frail families, represents a priority;
- As regards hospitalized pediatric cases, given the scarcity of data available in the literature, it would be advisable for pediatricians to identify a set of variables to be monitored over time using a shared methodology and definitions.

## Figures and Tables

**Table 1:** Preventive measures currently recommended in pediatric age in relation to SARS-CoV-2 transmission modes.

Viral Transmission Mode	Prevention	Level of Assistance
Interhuman (for close contact)	Social distancing of various degrees until the general closure	Blocking the transmission routes of the virus
Direct (respiratory secretions)	Physical distancing (interpersonal distance of at least one meter even during recreational/sports activity) Face mask (nose and mouth cover) in crowded or poorly ventilated environments and whenever it is not possible to maintain distancing, of adequate size. The WHO recommends its use from the age of 5 years, and the CDC already from 2 years of age in the absence of disability, considering it effective and safe	
Indirect (contamination of surface objects, aerosolization)	Proper and regular hand washing with soap and water or an alcoholic product Frequently cleaning surfaces Frequent aeration	
Infected (symptomatic and asymptomatic)	IsolationTracking close contacts (from 48 h before symptoms appear)	Control of the source of infection
Close contacts of infected subjects	Quarantine and active surveillance	Protection of the sensitive population
All susceptible subjects	Vaccination (as soon as available)	

**Table 2:** Usefulness and limits of antigenic and serological tests in pediatric age.

**Usefulness of Serological Tests**	**Usefulness of Antigen Testing**
Evaluation of patients with a high clinical suspicion of COVID-19 when the molecular test is negative and at least two weeks have passed since the onset of symptoms Monitoring of multisystemic inflammatory syndrome in children Conducting serosurveillance studies Potential utility to track the course of the SARS-CoV-2 pandemic in the community	Use as a screening test in high-risk environments Use in contexts where a rapid response time is required Use in environments with reduced ability to perform RT-PCR tests for logistical or economic reasons, being less expensive and easier to use
**Limits of Serological Tests**	**Limits of Antigen Testing**
Poor diagnostic utility in acute phase of infection Inability to be used in determining immunological status due to lack of data on efficacy and duration of anti-SARS-CoV-2 antibody response	Overall sensitivity lower than RT-PCR-based tests Poor diagnostic utility after 5 days of onset of symptoms

**Table 3:** Multisystemic inflammatory syndrome (MIS-C) classifications.

World Health Organization (WHO)	Royal College of Pediatrics and Child Health (RCPCH)	Center for Disease Control and Prevention (CDC)
Child or adolescent aged 0–19 years with fever >3 days and 2 of the following characteristics: rash or non-purulent conjunctivitis or signs of mucocutaneous inflammation (oral cavity, hands or feet) hypotension or shock signs of myocardial dysfunction, pericarditis, valvulitis or coronary abnormalities (including ultrasound alterations or troponin elevation /NT-proBNP) evidence of coagulopathy (PT, APTT, D-dimer elevation) acute gastrointestinal problems AND Elevation of inflammation indices such as CRP, PCT or ESR AND Exclusion of other microbiological causes of inflammation including bacterial sepsis, staphylococcal or streptococcal toxic shock syndrome: AND Evidence of SARS-CoV-2 infection (antigenic test or positive serology) or contact with COVID-19 patient Consider MIS-C in patients with typical/atypical Kawasaki disease or toxic shock syndrome	Patient with persistent fever (>38.5 °C), systemic inflammation (neutrophilia, PCR elevation and lymphopenia) and evidence of single or multiple organ dysfunction (shock, heart, kidney, gastrointestinal or neurological disorders) with additional characteristics * Patients with symptomatology partially or wholly meeting the criteria of Kawasaki’s disease may be included Exclusion of any other microbiological cause including bacterial sepsis, staphylococcal or streptococcal toxic shock syndrome, other infectious causes of myocarditis Searching for SARS-CoV-2 using PCR can be positive or negative* Additional features: Clinical: Many: O_2_ request, hypotension Some: abdominal pain, confusion, conjunctivitis, cough, diarrhea, headache, lymphadenopathy, changes in the mucous membranes, nuchal stiffness, rash, respiratory symptoms, pharyngitis, edema of the feet and hands, syncope, vomiting Laboratory: All: alteration of fibrinogen, high D-dimer, high ferritin, hypoalbuminemia Many: acute kidney damage, anemia, thrombocytopenia, coagulopathy, elevation IL- 10, -6, proteinuria, CK and LDH elevation, triglyceride elevation, troponin and liver transaminases Imaging: Echocardiography and ECG: myocarditis, valvulitis, pericardial effusion, dilation of the coronary arteries Radiography: Symmetrical pulmonary infiltrations, pleural effusion Abdomen echo: colitis, ileitis, lymphadenopathy, ascites, hepatosplenomegaly Pulmonary CT with contrast may show coronary aneurysms	Patients aged <21 years who have fever, laboratory evidence of inflammation and clinical evidence of severe prostration that requires hospitalization and the presence of two or more affected organs/apparatuses (heart, kidney, respiratory system, hematopoietic, gastrointestinal, dermatological or neurological) Fever >38 °C for ≥24 h or subjective fever reported for more than 24 h Laboratory positivity of more than 1 of the following indices: CRP, ESR, PCT, fibrinogen, D-dimer, ferritin, LDH or IL-6; neutrophilia, lymphopenia and hypoalbuminemia AND No other plausible diagnoses AND Laboratory positivity for recent or ongoing infection for SARS-CoV-2 (positivity of molecular, antigenic or serological investigations or contact with a certain case of COVID-19 in the previous 4 weeks) Comment Patients who partially or wholly meet the criteria of Kawasaki’s disease should be reported if they meet the MIS-C criteria Consider MIS-C in pediatric death cases with evidence of SARS-CoV-2 infection

APTT, activated partial thromboplastin time; CRP, C reactive protein; CT, computed tomography; ECG, electrocardiogram; ESR, erythrocyte sedimentation rate; IL, interleukin; LDH, lactate dehydrogenase; MIS-C, multisystemic inflammatory syndrome; NT-proBNT, N-terminal pro b-type natriuretic peptide; PCT, procalcitonin; PT, prothrombin time.

**Table 4:** Laboratory/instrumental tests in progress of multisystemic inflammatory syndrome (MIS-C).

Blood Tests	Hemochrome with formula: leukocytosis with lymphopenia. In case of leukopenia, platelets or anemia, consider the hypothesis of sHLH/MAS CRP, ESR and PCT: highFerritin: high. In case of low ESR with high CRP or hyperferritinemia, consider the sHLH/MAS hypothesis Coagulation: high fibrinogen, high D-dimer. Evaluate PT and PTT for any pro-thrombotic alterations. In case of hypofibrinogenemia, consider the hypothesis of sHLH/MAS. Electrolytes: possible hyponatremia Liver function: in case of hypertransaminasemia, consider the hypothesis of sHLH/MAS. Cases of MIS-C with gallbladder hydrops are described, which may occur with hyperbilirubinemia Kidney function: cases of MIS-C with kidney failure are described Troponin and ProBNP: high in case of heart attack. Total protein, Seric albumin: hypoalbuminemia may be present Triglycerides: in case of hypertriglyceridemia, consider sHLH/MAS CPK, LDH: they can be increased, highlight the possible cytolysisIL- 6: high C3, C4: hypocomplementemia may be present for consumption γGT: in combination with the other liver function tests (first level) may testify to any hepatopathy Pancreatic functionality: cases with increased lipase and amilase are described Urine examination: leukocyturia may be present in the absence of typical elements for urinary tract infection, signs of tubular damage. Hemogasanalysis: metabolic acidosis. Increase in infants Peripheral smear: the presence of schistocytes or Burr cells, which testify to microangiopathy, is described.
Microbiological Examinations In most cases, the main differential diagnosis is sepsis.	Hemoculture, urinoculture, coproculture Serologies In case of positive serology, where available, confirmation with CRP Nasopharyngeal aspirate for influenza A and B, RSV and adenovirus is useful
Instrumental Examinations	ECG + Echocardiogram: hypokinesis in case of myocarditis, valve failure from myocarditis, pericarditis, coronary artery changes. In case of a patient with shock, it can also be useful to evaluate the state of hydration. RX chest: interstitial pneumonia. Pleurisy and increased pericardial effusion heart shadow may be present. Abdomen ultrasound: presence of organomegaly, peritoneal effusion, hydrops of the gallbladder Chest CT: in case an in-depth study of lung, locating is necessary Cardiac MRI: it can be useful, where available, in case of proven myocarditis Colonoscopy: in selected cases with severe intestinal involvement

C, complement; CPK, creatine phosphokinase; CRP, C reactive protein; CT, computed tomography; ECG, electrocardiogram; ESR, erythrocyte sedimentation rate; γGT, gamma-glutamyltransferase; LDH, lactate dehydrogenase; MIS-C, multisystemic inflammatory syndrome; MRI, magnetic resonance imaging; PCT, procalcitonin; PCT, procalcitonin; proBNP, pro b-type natriuretic peptide; PT, prothrombin time; PTT, partial thromboplastin time; RX, radiography; RSV, respiratory syncytial virus; sHLH/MAS, macrophage activation syndrome.

**Table 5:** Proposal for immunomodulation in multisystemic inflammatory syndrome (MIS-C).

IMMUNOGLOBULINS First-line therapy	IVIG 2 gr/kg (calculation based on ideal weight) in a single administration in at least 12 h. In the case of a patient with pump deficiency/alteration of the water balance, the IVIG should be administered in at least 16–24 h, or alternatively the hypothesis of splitting the total dose into two administrations should be considered. In the event of ineffectiveness/poor response, the appropriateness of administering a second dose should be considered.
GLUCOCORTICOIDS Recommended in combination with the first line of high-risk therapy according to the protocol of Kawasaki disease (age < 12 months, heart failure, coronary artery change). Second line of therapy in case of failure of the first line of therapy together with 2° bolus of IVIG. The association between IVIG and corticosteroids (2 mg/kg methylprednisolone) may be an option to consider for all cases of MIS-C, taking into account that in similar models, the combination of the two treatments seems to reduce the risk of complications.	(A) Methylprednisolone 2 mg/kg i.v. in 3 doses/day with decalage in 2–3 weeks (B) Methylprednisolone 10–30 mg/kg up to a maximum of 1 g in bolus i.v. once a day for 1–3 days, followed by methylprednisolone/prednisone 2 mg/Kg/day with decalage in 2–3 weeks The choice of A or B should be assessed on the basis of the severity of the clinical picture and/or laboratory picture. In particular, in the case of clinical/laboratory elements of sHLH/MAS or in case of shock, the use of high-dose iv steroids seems reasonable. In case of signs of CNS involvement, it is useful to consider the use of dexamethasone (10 mg/m^2^/die)
BIOLOGICAL DRUGS (anakinra, tocilizumab, infliximab) Third line of therapy: in case of poor response to IVIG and steroid therapy First/second line of therapy: in case of a particularly severe clinical picture even at onset (e.g., signs of sHLH/MAS, shock or myocarditis with severe pump deficiency) in clinical judgment	Anakinra (Kineret: 2 mg/kg × 4/day max 100 mg i.v.) diluted in physiological solution and administered at max 1 h from the preparation-vd appendix or in continuous infusion at the dose of 2 mg/kg attack dose (in bolus) followed by a total dose of up to 12 mg/kg/day (anakinra vial has a stability of about 6 h) for a maximum daily dose of 400 mg. Anakinra s.c.: 2–6 mg/kg/day Tocilizumab i.v.: <30 kg 12 mg/kg ev, >30 kg: 8 mg/kg Infliximab: 5 mg/kg/day in 200–500 mL of physiological solution in 2 h i.v.

CNS, central nervous system; IVIG, intravenous immunoglobulin; sHLH/MAS, macrophage activation syndrome.

**Table 6:** Complementary therapies in multisystemic inflammatory syndrome (MIS-C).

COMPLEMENTARY THERAPIES	✓ Broad-spectrum antibiotic coverage pending the result of cultures (preferring association with clindamycin) • Proton pump inhibitor • Cardiovascular support • Acetylsalicylic acid at antiaggregating dosage: 3–5 mg/kg/day in a single administration per os for at least 6–8 weeks ✓ Prophylaxis/antithrombotic therapy • In patients with thromboembolic risk factors or D-dimer elevation: Anti-thrombotic prophylaxis: low-molecular weight heparin 2 times/day (anti-X target activated 4 h from heparin somministration: 0.2–0.5 U/mL) In the case of venous thrombosis or coronary aneurysms *, systolic ventricular dysfunction: Anti-thrombotic therapy in medical judgment/reference hemostasis center * Coronary aneurysms are given treatment guidance according to the *American Heart Association* In case of acute renal failure and schistocytes, the hypothesis of acute uremic-hemolytic syndrome/thrombotic microangiopathy and the possible use of eculizumab iv should be considered

**Table 7:** Risk factors for thrombosis.

Central venous catheter Mechanical ventilation Prolonged immobilization Obesity (Body Mass Index >95° percentile) Tumors, nephrotic syndrome, exacerbation of cystic fibrosis, falcemic vaso-occlusive crisis, autoimmune or inflammatory underlying disease Congenital or acquired cardiopathy with venous stasis or altered venous return Personal or family history of thromboembolic events Known Thrombophilia (Protein S, Protein C, Antithrombin Deficiency, Leiden V Factor; Factor II G20210A; Anti-Phospholipid Antibodies) Puberal or post-puberal age (>12 years) Estrogen or estrogen–progestogen therapy Splenectomized patients for hemoglobinopathy

**Table 8:** Stratification of thromboembolic risk in patients with multisystemic inflammatory syndrome (MIS-C) in relation to D-dimer values.

	D-dimer ≥5 Times Normal Values	Additional Risk Factors	Suggested Anticoagulation
Patient with MIS-C	YES	NO/ND	YES
	NO	One or more	YES
	NO	NO	NO

## Data Availability

Data sharing not applicable. All details are presented in the Methods section and the text has been written on findings reported in the mentioned References.
